# A Point Mutation V419L in the Sodium Channel Gene from Natural Populations of *Aedes aegypti* Is Involved in Resistance to λ-Cyhalothrin in Colombia

**DOI:** 10.3390/insects9010023

**Published:** 2018-02-14

**Authors:** Yurany Granada, Ana María Mejía-Jaramillo, Clare Strode, Omar Triana-Chavez

**Affiliations:** 1 Grupo Biologia y Control de Enfermedades Infecciosas—BCEI, Universidad de Antioquia, Calle 70 No. 52-21, Medellín 050010, Colombia; eresbey2@gmail.com (Y.G.); anamejia25@gmail.com (A.M.M.-J.); 2 Biology Department, Edge Hill University, St. Helens Road, Ormskirk, Lancashire L39 4QP, UK; strodecl@edgehill.ac.uk

**Keywords:** *Aedes aegypti*, insecticide resistance, knockdown resistance (kdr), sodium channel gene

## Abstract

Resistance to pyrethroids in mosquitoes is mainly caused by target site insensitivity known as knockdown resistance (*kdr*). In this work, we examined the point mutations present in portions of domains I, II, III, and IV of the sodium channel gene in *Aedes aegypti* mosquitoes from three Colombian municipalities. A partial region coding for the sodium channel gene from resistant mosquitoes was sequenced, and a simple allele-specific PCR-based assay (AS-PCR) was used to analyze mutations at the population level. The previously reported mutations, V1016I and F1534C, were found with frequencies ranging from 0.04 to 0.41, and 0.56 to 0.71, respectively, in the three cities. Moreover, a novel mutation, at 419 codon (V419L), was found in *Ae. aegypti* populations from Bello, Riohacha and Villavicencio cities with allelic frequencies of 0.06, 0.36, and 0.46, respectively. Interestingly, the insecticide susceptibility assays showed that mosquitoes from Bello were susceptible to λ-cyhalothrin pyrethroid whilst those from Riohacha and Villavicencio were resistant. A positive association between V419L and V1016I mutations with λ-cyhalothrin resistance was established in Riohacha and Villavicencio. The frequency of the F1534C was high in the three populations, suggesting that this mutation could be conferring resistance to insecticides other than λ-cyhalothrin, particularly type I pyrethroids. Further studies are required to confirm this hypothesis.

## 1. Introduction

*Aedes aegypti* (L.) is the main vector for Zika, dengue, and chikungunya viruses. The diseases caused by these viruses are a rapidly growing public health problem worldwide, due to an increase in the mosquito’s population, to rapid human urbanization with population growth, climate parameters, and human migration [1,2]. During 2016, there was an alarming number of new cases for Zika, dengue, and chikungunya in Colombia, with incidences around 100,000, 83,000, and 23,000 cases, respectively [3].

With no suitable vaccines currently available, strategies to control these diseases are focused on reducing the mosquito population through the use of insecticides. In Colombia, these strategies have been mainly based on using insecticides such as DDT, temephos, malathion, and, more recently, pyrethroids [4]. Unfortunately, most dengue vector control programs underway are poorly implemented, resulting in the development of insecticide resistance. Insecticide resistance in mosquitoes is caused by a number of mechanisms, with two in particular being the focus of the majority of studies: metabolic resistance and target site alterations. Metabolic resistance involves large multi-gene enzyme families: cytochrome P450s, glutathione S-transferases, and carboxyl esterases. Cytochrome P450s, in particular, have been implicated in conferring resistance in mosquitoes [5,6]. The role of cuticular resistance and behavioral avoidance of insecticides are less studied. Target site insensitivity, known as knockdown resistance (*kdr*), where several point mutations in the gene encoding the sodium channel have been associated with resistance to pyrethroids. To date, at least 10 resistance-associated sodium channel mutations have been identified in pyrethroid-resistant *Ae. aegypti* and four of them, S989P, I1011M, V1016G, and F1534C, have been functionally confirmed to confer sodium channel resistance to pyrethroids [6,7]. The mutations G923W, L982W, I1011M, and V1016G in domain II, were found in permethrin/DDT-resistant populations from Asia and Brazil [8], while substitutions I1011V and V1016I were found in *Ae. aegypti* populations from Latin America [9]. Also, the F1534C substitution in domain III-S6 was discovered in Vietnam and Thailand [10,11,12], and more recently in Brazil [13], Venezuela [14], and Colombia [15]. Finally, two additional mutations, S989P and D1763Y, were found in Asiatic countries [16,17]. In addition, double mutations, V1016G and F1534C, were reported in Singapore [18] and triple mutations S989P, V1016G, and F1534C in *Ae. aegypti* from China, Indonesia, and Thailand [11]. In America, the mutation V1016I was discovered to coexist with F1534C in Venezuela [14] and Brazil [19,20,21]. Interestingly, Vera-Maloof et al., in 2015, proposed that the F1534C mutation was selected first and confers a low level of pyrethroid resistance, and that the V1016I haplotype cannot be selected in the absence of F1534C [22].

Regular monitoring has revealed that many Colombian populations of *Ae. aegypti* have become resistant to pyrethroid insecticides [23,24,25,26]. However, the underlying mechanism for this resistance is not yet known. Recently, a valine to isoleucine substitution at position 1016 (V1016I) within the second domain of the *Ae. aegypti* sodium channel was observed in Colombian Caribbean populations with allelic frequencies ranging from 0.07 to 0.35 [4] and 0.03 [27]. Unfortunately, these last studies only investigated mutations previously reported in mosquitoes from other countries around the world. Thus, the identification of known and novel mutations present in *Ae. aegypti* populations from Colombia and their association with insecticide resistance should be studied in more detail.

Therefore we aimed to measure resistance level to λ-cyhalothrin and to investigate the association between *kdr* mutation and resistance to this insecticide in mosquitoes from three Colombian cities that have different epidemiological characteristics for dengue. For this, the partial coding region of the sodium channel gene from resistant mosquitoes was sequenced and a simple allele-specific PCR-based assay (AS-PCR) was used to detect these mutations at the population level. We report a new mutation in the coding region of the sodium channel from wild mosquitoes involved in the resistance to this pyrethroid.

## 2. Materials and Methods

### 2.1. Mosquito Collection

**Figure 1:**
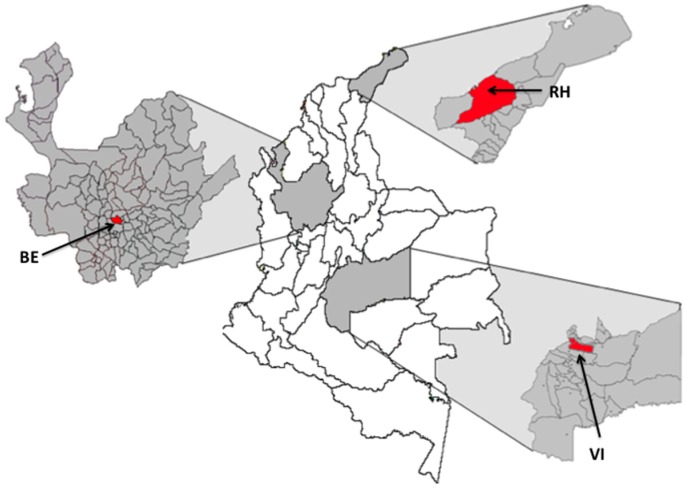
Map of Colombia showing cities where samples of *Aedes aegypti* mosquitoes were collected. BE: Bello, RH: Riohacha, and VI: Villavicencio.

Both adult mosquitoes and immature stages were collected during 2013–2014 in three cities of Colombia with the assistance of staff involved in vector-borne diseases programs for each municipality. Larvae and pupae were collected from the deployment of ovitraps, and adults were collected using sweep nets, in 20 randomized houses from four neighborhoods [28]. The cities sampled are localized in three eco-geographical regions showing distinct weather and dengue incidence, as was reported previously [28]. Bello (BE), located in the central Andean region of Colombia at an altitude of 1250 m, is characterized by the reduced use of chemical insecticides, while Riohacha (RH) and Villavicencio (VI), located at an altitude of 2 and 467 m, respectively, are frequently exposed to vector control using chemical insecticides (Figure 1).

The adult mosquitoes were individually placed in absolute ethanol (99.8%) and stored in a freezer at −20 °C until molecular analysis was performed. The immature stages were reared until adults emerged and maintained under controlled conditions of temperature (28 ± 1 °C), relative humidity (80 ± 5%), and photoperiod (12 h light: 12 h dark).

### 2.2. Insecticide Bioassays

The insecticide being evaluated was the pyrethroid λ-cyhalothrin. Around 20 *Ae. aegypti* third- or fourth-instar F1 larvae, were exposed to five concentrations of the insecticide (0.0031 ppm to 0.5 ppm) to determine larval mortality 24 h after exposure, following the methods proposed by the World Health Organization (WHO) [12]. The insecticide susceptible Rockefeller strain (of Caribbean origin, that has been in colony since the early 1930s [29,30]) (kindly provided by Grupo de Sistemática Molecular of Universidad Nacional de Colombia), was used as a control. Three replicates were performed at each concentration and control for each wild population and the Rockefeller strain.

The lethal concentrations (LC_50_) and confidence intervals (*p* < 0.05) were calculated for every mosquito population. Concentration-mortality data were subjected to Probit analysis and 95% CIs for resistance ratio (RRs) were estimated. A resistance ratio (RR50) was obtained by dividing the lethal concentration of the population by the equivalent lethal concentration of the Rockefeller reference strain. The RR was interpreted as susceptible (<5-fold), tolerant or moderate resistance (5- to 10-fold) and high resistance (>10-fold) [18]. *p*-values < 0.05 were considered statistically significant.

### 2.3. RNA Extraction and cDNA Synthesis

The surviving larvae to λ-cyhalothrin treatment were reared to adults, and total RNA was extracted using Qiagen’s Trizol kit (QIAGEN, Duesseldorf, Germany). cDNA synthesis was performed using 2 μg of RNA, 0.5 μg oligo dT and retrotranscriptase Biolab (New England BioLabs Inc., Ipswich, MA, USA). The cDNA was stored to −20 °C.

### 2.4. cDNA Sequencing

In order to identify the mutations present in the sodium channel gene, we amplified the partial coding region of this gene from Villavicencio population (with the highest resistance ratio) and Rockefeller strain (reference susceptible). Portions of domains IS4-S6, IIS1-S6, and IIIS4-S6 of this gene were amplified using the primer sets previously reported by Yanola et al., 2011 [12]. The PCR products were purified and then sequenced by Macrogen Inc. (Seoul, South Korea). The sequences were edited using the Biological Sequence Alignment Editor (BioEdit version 7.2.1, Ibis Biosciences, Carlsbad, CA, USA) program, and aligned using the MEGA5 program, with the aim of identifying the mutations present in this region. The sequences were also compared with others available on GenBank using tblastx to verify the amplified fragment and identify the point mutations.

### 2.5. Allele-Specific PCR (AS-PCR) Genotyping

A minimum of 57 adult mosquitoes from each city was processed for genotyping. Genomic DNA from individual mosquitoes was extracted using the protocol described by Collins et al. [31]. The samples were genotyped at positions 1255, 3046, and 4673 of the sodium channel gene using allele-specific PCR (AS-PCR). For this, we designed an allele-specific primer to 419 mutation (G1255T); and we used allele-specific primers previously developed to 1016 (G3046A) and 1534 (T4673G) mutations [12,32] (Table 1). Each reaction was performed in a 25 μL volume consisting of 4.5 mM MgCl_2_, 1X PCR buffer (Thermo Scientific, Waltham, MA, USA), 0.3 μM of each primer, 200 μM dNTP mixture, 0.16 units Taq polymerase, and 20 ng genomic DNA. The amplification reactions were performed with an initial denaturation step of 3 min at 94 °C, followed by 35 cycles of 30 s at 94 °C, 30 s at 60 °C, and 60 s at 72 °C, with 7 min at 72 °C for final extension, according to the KAPA kit instructions (Kapa Biosystems, Boston, MA, USA). PCR amplification products were separated on a 2% agarose gel in a TE buffer, at 100 V for 60 min. The Rockefeller strain of *Ae. aegypti* was used as a reference for the wild-type alleles (419L, 1016V and 1534C) of the sodium channel gene. The allelic frequencies were calculated from the gel results by summing the genotypic frequency of the heterozygotes divided by two and the frequency of the homozygotes. GENEPOP v.4.6 (Laboratoire de Genetique et Environment, Montpellier, France, http://genepop.curtin.edu.au/) was used to calculate allelic and genotypic frequencies and the data were tested for conformity to Hardy– Weinberg equilibrium [33].

### 2.6. Correlation of Resistance with the Frequencies of Different Mutations

To evaluate whether the frequency of point mutations observed in Colombian populations was correlated with resistance to λ-cyhalothrin, a descriptive analysis was conducted comparing the *kdr* allele frequency and the resistance ratio values. In order to verify the role of these mutations in the resistance to λ-cyhalothrin, mosquitoes from Villavicencio were maintained in the laboratory without insecticide pressure until F8 filial generation. The allelic frequencies in 52 mosquitoes were also calculated.

## 3. Results

### 3.1. λ-Cyhalothrin Bioassays and Adult Genotyping

The mosquitoes from the three cities presented different levels of resistance to λ-cyhalothrin. The resistance ratios (RR) ranged from 1.97 for mosquitoes from Bello to 10.96 and 11.34-fold for mosquitoes from Riohacha and Villavicencio, respectively. Table 2 shows the values of LC_50_ and RR obtained for this insecticide.

### 3.2. Comparison of cDNA Region from Susceptible and Resistant Mosquitoes and Identification of Sodium Channel Gene Mutations

**Figure 2:**
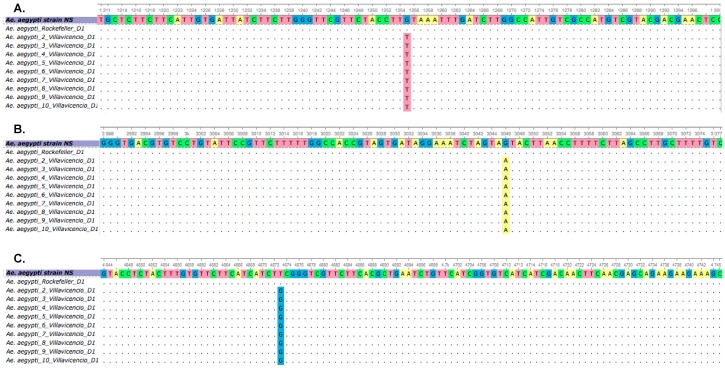
Alignment of cDNA fragments of the DIS4-6 (**A**), DIIS1-6 (**B**) and DIIIS4-6 (**C**) regions of the sodium channel gene obtained for *Ae. aegypti* from Villavicencio and Rockefeller strain. The sequences were aligned with ClustalW version 1.81. Identical nucleotides are indicated by a dot (.). The sequences were also compared with *Ae. aegypti* strain NS available from GenBank and one mosquito from Rockefeller strain, susceptible to pyrethroids. The resistant mosquitoes were 100% identical. (**A**): point mutation G1255T (V419L), (**B**): mutation G3046A (V1016I) and (**C**). Mutation T4673G (F1534C).

The mosquitoes with highest RR were analyzed for the presence of mutations. The partial coding region of the sodium channel gene from nine resistant mosquitoes was sequenced and aligned with sequences from susceptible mosquitoes (Figure 2). Interestingly, all the mosquitoes analyzed contained the three point mutations, two of which, V1016I and F1534C, have been previously reported, whilst the novel mutation V419L, is being reported here for the first time in Colombian mosquitoes. This new mutation was identified in the domain I of segment S4–S6 (IS4–6) from resistant mosquitoes of Villavicencio (Figure 2). All three mutations were used to design an AS-PCR to identify the genotypic and allelic frequencies at the population level.

### 3.3. AS-PCR for the V419L, V1016I and F1534C Mutations

**Figure 3:**
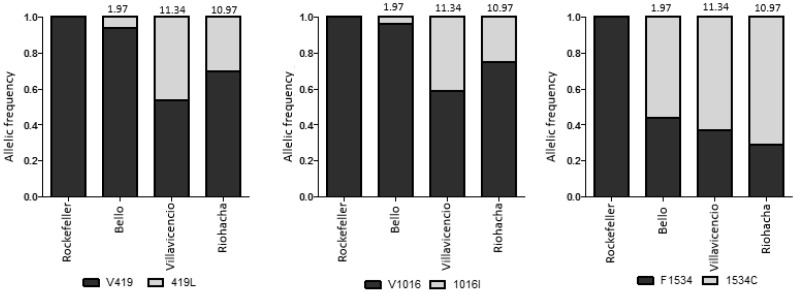
Comparison of allelic frequencies for the mutations 419L, 1016I and 1534C in *Aedes aegypti* from three cities studied in Colombia. The RR value for mosquitoes from each city is shown at the top of each column.

In order to determine the frequency of three mutations in each city, a minimum of 57 mosquitoes were genotyped using AS-PCR (Figure S1). The genotypic frequencies of V419L, V1016I and F1534C mutations are shown in Table 3. For the V419L mutation, all three genotypes were observed (V/V, V/L and L/L) in the three cities, although the heterozygote (V419/419L) and homozygote (419L/419L) genotypes were rare in the Bello municipality. Similarly, the 419L mutant allele frequency was found at 0.06 in Bello, whilst in Villavicencio and Riohacha, was 0.46 and 0.30, respectively (Figure 3). All three genotypes at position 1016 were observed in the three cities (V/V, V/I and I/I), and the 1016I mutant allele frequency in Villavicencio and Riohacha, was 0.41 and 0.25, respectively, in comparison to 0.04 observed in Bello. Conversely, there was no clear difference in the frequency of allele 1534C, between mosquitoes from the three cities with values of 0.63, 0.71 and 0.56 in Villavicencio, Riohacha and Bello, respectively. All genotype frequencies across the three different populations conformed to Hardy–Weinberg equilibrium (Table 3). A comparative analysis between allelic frequencies and resistance ratio of these three alleles in the three cities studied is shown in Figure 3.

### 3.4. Correlation of Resistance with the Frequencies of the Different Mutations

**Figure 4:**
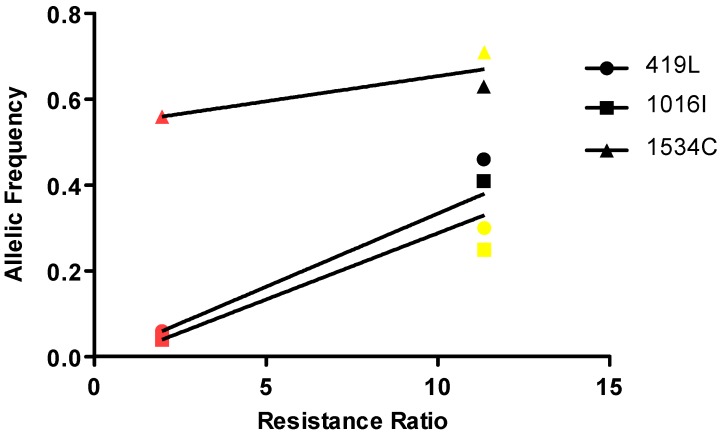
Relationship between resistance ratio (RR) to λ-cyhalothrin and frequency of *kdr* alleles (419L, 1016I and 1534C) in *Aedes aegypti* from Colombia. The red, yellow and black symbols represent Bello, Riohacha and Villavicencio mosquito populations, respectively.

A positive correlation between the frequency of the 419L and 1016I alleles and resistance to λ-cyhalothrin was observed. Mosquitoes from Bello presented low frequency of these alleles. In contrast the frequency of the 1534C mutation was not correlated with the resistance to λ-cyhalothrin (Figure 4). Mosquitoes from Bello, Villavicencio and Riohacha showed a high frequency of the 1534C mutated allele (0.56, 0.63 and 0.71, respectively).

### 3.5. Mutations in Combination

Individuals with heterozygote genotypes for 419L and 1016I positions were found at high frequency in mosquitoes from Villavicencio and Riohacha. These double heterozygotes were observed together with the mutation F1534C (Haplotype V419L/V1016I/1534C). Triple heterozygotes (V419L/V1016I/F1534C) and triple mutant homozygotes (419L/1016I/1534C) were observed at low frequency in the three cities. Mosquitoes with only the point mutation 419L were observed in Riohacha (Table 4).

### 3.6. Allelic Frequencies in Absence of Insecticide

For the V419L mutation, 76.92% of mosquitoes presented the wild genotype in comparison with the 32.91% observed in the parental population. In the F8 population, the homozygote-mutated genotype was not observed. The same situation was observed for the 1016I mutation, with a change of 26.96% to 76.92%. Conversely, for the 1534 mutation, the homozygote-mutated genotype allelic frequencies were not significantly affected (a change of 0.94 to 0.83).

## 4. Discussion

*Aedes aegypti* has been reported in around 700 municipalities from the 32 departments in Colombia [34]. Notably the cases of arboviruses transmitted by this vector have been remained stable or have increased over time, which is probably due to the broad distribution of this mosquito. The main way of preventing virus transmission is by using chemical insecticides. In order to design well-structured and successful insecticide-based control programs, it is important to know the insecticide resistance status of *Ae. aegypti* in the different regions of the country. Molecular surveillance of insecticide resistance is an important component for the design of effective control campaigns.

In this study, we assessed the levels of resistance to λ-cyhalothrin from three Colombian cities. Riohacha and Villavicencio are cities where use of insecticides is widespread; whilst in Bello they are rarely used. DNA sequencing of domains I, II and III of the sodium channel gene from resistant mosquitoes collected in Villavicencio revealed the presence of three point mutations. To establish an association between *kdr* mutations and pyrethroid resistance, we compared the resistance ratio with the allelic frequencies of each mutation observed in these populations. Interestingly, the resistant mosquito populations (Villavicencio and Riohacha) presented the V1016I mutation at a higher frequency than susceptible population (Bello). The 1016I allele was reported for the first time in Colombia in 2014, but no association with resistance to λ-cyhalothrin was found [4]. Recently, Aguirre-Obando et al. found that this mutation was present at a low frequency in mosquito populations from Colombia with susceptibility to temephos [27]. Together these results suggest that this allele is circulating in Colombian mosquito populations, and the uncontrolled use of insecticides could increase the frequency of this allele. Moreover, we have demonstrated a clear difference of allele 1016I between resistant and susceptible populations. In line with our results, a V1016G mutation was positively correlated with resistance to pyrethroids in *Ae. aegypti* populations from China [32], and this mutation appears to be the most important *kdr* mutation in this region. Our results provide strong evidence that the mutation V1016I also contributes to pyrethroid resistance. However, Du et al. found that V1016I did not alter channel sensitivity when they tested the functionally of this mutation in *Xenopus*, whilst V1016G mutation did have a significant functional effect [35]. Further studies are therefore needed to demonstrate the role of the 1016I allele.

The frequency of the 1534C allele in mosquito populations from Villavicencio, Riohacha, and Bello was 0.63, 0.71, and 0.56, respectively. There was no clear difference in the frequency of the 1534C allele between resistant and susceptible mosquito populations. This result suggests that this mutation apparently does not have a resistance effect with λ-cyhalothrin, although some studies revealed that this mutation reduces the sensitivity to permethrin [36,37]. Interestingly, permethrin is a type I pyrethroid, while λ-cyhalothrin belongs to type II pyrethroids; this could explain why this mutation, despite being at a high frequency in the Bello population, did not correlate with resistance.

We report for the first time the V419L mutation in natural populations of *Ae. aegypti* from Colombia. This mutation was positively correlated with resistance to λ-cyhalothrin. The frequency of this mutation was closely related to the frequency of the mutation at position 1016, with values of 0.46, 0.30, and 0.06, for Villavicencio, Riohacha, and Bello, respectively. Recently, Haddi et al. (2017) reported the presence of this mutation in resistant mosquitoes from Brazil, and this mutation together with mutation F1534C increased strongly the resistance to both type I and II pyrethroids [38]. This result suggests that domain I has a significant effect on the resistance to these insecticides. New studies using functional genomics should be performed to describe the role of V419L mutation in the sodium channel function. In light of our current findings and those of Haddi et al. (2017), we propose that V419L mutation should be taken into account in future studies investigating target site mutations in response to the use of pyrethroids.

A number of mosquitoes in this study harbored only one mutation, and others presented two or three mutations. Interestingly, the V419L mutation was found more frequently alone in some individuals, indicating that it is the first to appear in these mosquitoes. This result does not support those reported by Vera-Maloof et al., which indicated that F1534C mutation was selected first [22]. In addition, our results support the origin of this mutation occurring in independent events and on separate haplotypes. Future studies are required to evaluate if this mutation originated in each population or if it was introduced to Colombia. On the other hand, the additive effect of *kdr* mutations on resistance to pyrethroids, have been previously reported [11,35,39]. The mutations 1016G and 1534C increased the resistance to deltamethrin compared with 1534C homozygote individuals [40]. Likewise in mosquito populations from Asia, the mutations V1016G and F1534C have been reported separately in two cities [16]. However, in Mexico, both mutations (V1016I and F1534C) appear in the same haplotype, with subsequent strong selection for the linked mutations [22]. The effect of the double mutations V419L and V1016I therefore requires further study. 

Different genotypes observed between cities are probably due to the populations having different genetic backgrounds and/or being exposed to differential usage of insecticides. The first option is not supported by microsatellite studies as they show that there is genetic flow between the mosquitoes in the three cities [41]. The second option is therefore more likely. Riohacha and Villavicencio have higher incidences of dengue, Zika, and chikungunya than Bello and consequently insecticides are used more widely in these two cities. The differences between the allelic frequencies indicate that selection pressures on *Ae. aegypti* are occurring at different levels in these cities.

In conclusion, we propose that the observed resistance to λ-cyhalothrin by the populations of *Ae. aegypti* from Villavicencio and Riohacha is mediated by the presence of mutations at positions 419 and 1016. Recently, Ponce-García et al., reported that the F1534C and V1016I mutations are widespread in Puerto Rico populations of *Ae. aegypti*, but they are not involved in resistance to permethrin [42]. It is likely that other mutations such as V419L and/or other mechanisms such as metabolic resistance could be involved in these populations.

We are aware that, while evidence of *kdr* has been found, we cannot discount the role of other resistance mechanisms conferring the phenotype. Most notable is metabolic resistance, such as the involvement of cytochrome P450s, which have been shown to play a role in conferring resistance in both *Ae. aegypti* and other mosquito vectors particularly *Anopheles gambiae* [5,19,38,43,44]. We are currently using transcriptomics to assess the role of metabolic resistance and other mechanisms, including cuticular resistance, in resistant populations from Colombia.

Finally, the indiscriminate use of insecticides to control mosquito populations has led to the development of resistance. Therefore, it is vital to carry out continuous and appropriate monitoring of the presence of these mutations and other resistance mechanisms associated with the loss of susceptibility in populations of *Ae. aegypti.* This would allow for the implementation of an effective control program that could counteract the presence of arboviruses transmitted by this mosquito in Colombia. For successful dengue, chikungunya, and Zika control, two key elements are needed. Firstly, it is important to know the status of resistance in different regions of the country to design bespoke and well-structured strategies. Secondly, it is important to provide training for the poorest communities that are most at risk and in time design sustainable control strategies for these communities.

## 5. Conclusions

A novel mutation, V419L, was found in *Ae. aegypti* from Colombia. A significant association was found between this mutation and pyrethroid resistance, as well as the mutation V1016I. The allele F1534C occurred frequently in the three populations, probably indicating that this mutation could be conferring resistance to other insecticides. Further studies are required to confirm this hypothesis.

## Figures and Tables

**Table 1:** Allele-specific primers used in genotyping of *Aedes aegypti* from Colombia.

Mutation	Primers	Sequence	Reference
V419L	PM1_Ext_419F	GATTCCTCCAGAACTCCACC	This paper
	PM1_Ext_419R	TCAATGGATTTGGGTGACAA	
	PM1_F_419Wt	CTTGGGTTCGTTCTACCTTG	
	PM1_F_419Mut	CTTGGGTTCGTTCTACCTTT	
V1016I	PM2_Ext_1016F	GCCACCGTAGTGATAGGAAATC	Li et al., 2015 [32]
	PM2_Ext_1016R	CGGGTTAAGTTTCGTTTAGTAGC	
	PM2_F_1016Wt	GTTTCCCACTCGCACAGGT	
	PM2_F_1016Mut	GTTTCCCACTCGCACAGA	This paper
F1534C	PM3_Ext_1534F	GGAGAACTACACGTGGGAGAAC	Li et al., 2015 [32]
	PM3_Ext_1534R	CGCCACTGAAATTGAGAATAGC	
	PM3_R_1534WT	GCGTGAAGAACGACCCGA	
	PM3_R_1534Mut	GCGTGAAGAACGACCCGC	

**Table 2:** Resistance ratio to λ-cyhalothrin of *Aedes aegypti* populations studied in Colombia.

Mosquito Population	*n*	LC_50_	Confidence Limit	RR_50_	Phenotype
Rockefeller	360	0.01614	0.009–0.02323	-	-
Bello	300	0.03182	0.0282–0.0363	1.97	Susceptible
Villavicencio	300	0.1831	0.1320–0.2764	11.34	Resistant
Riohacha	300	0.1768	0.1220–0.2926	10.96	Resistant

**Table 3:** Genotype frequencies and associated *p* values for Chi-Square tests for deviation from Hardy–Weinberg equilibrium of sodium channel mutations in *Aedes aegypti* from Colombia.

	**Genotype (1016)**				**Hardy-Weinberg Parameters**			
**Location**	**V/V**	**V/I**	**I/I**	***n***	**Ho**	**He**	**X^2^**	***p***
**Villavicencio**	24	57	8	89	0.64	0.48	0.10	0.75
**Riohacha**	47	43	1	91	0.47	0.37	0.07	0.79
**Bello**	93	7	1	101	0.07	0.09	0.03	0.86
	**Genotype (1534)**				**Hardy-Weinberg Parameters**			
**Location**	**F/F**	**F/C**	**C/C**	***n***	**Ho**	**He**	**X^2^**	***p***
**Villavicencio**	9	32	16	57	0.48	0.47	0.06	0.81
**Riohacha**	7	22	34	63	0.35	0.41	0.02	0.89
**Bello**	9	32	16	57	0.56	0.49	0.02	0.89
	**Genotype (419)**				**Hardy-Weinberg Parameters**			
**Location**	**V/V**	**V/L**	**L/L**	***n***	**Ho**	**He**	**X^2^**	***p***
**Villavicencio**	26	33	20	79	0.42	0.50	0.03	0.86
**Riohacha**	32	24	7	63	0.38	0.42	0.01	0.92
**Bello**	52	5	1	58	0.09	0.11	0.06	0.81

*n*: number of mosquitoes analyzed; Ho: Heterozygosis observed; He: Heterozygosis expected.

**Table 4:** Haplotypes observed in *Aedes aegypti* mosquitoes populations from Villavicencio, Riohacha and Bello.

Haplotypes	Villavicencio	Riohacha	Bello
V419/V1016/F1534	0	4	7
V419/V1016/1534C	8	9	11
V419/V1016/F1534C	7	10	27
419L/V1016/F1534	0	1	0
V419L/V1016/F1534	0	1	0
V419L/V1016/1534C	2	0	0
V419L/V1016/F1534C	0	2	0
419L/1016I/1534C	6	1	1
V419/V1016I/1534C	4	4	0
V419/V1016I/F1534C	1	0	0
419L/V1016I/F1534	0	1	0
419L/V1016I/1534C	9	2	0
V419L/V1016I/1534C	20	12	2
V419L/V1016I/F1534C	3	6	3

## Supplementary Materials

The following are available online at http://www.mdpi.com/2075-4450/9/1/23/s1. Figure S1: Allele specific PCR profile representative of point mutations obtained in *Ae. aegypti* mosquitoes from three Colombian cities.
