# Supplementary material for: A Point Mutation V419L in the Sodium Channel Gene from Natural Populations of Aedes aegypti Is Involved in Resistance to λ-Cyhalothrin in Colombia
Source: Insects. 2018 Feb 14;9(1):23. doi: 10.3390/insects9010023 (PMC5872288; doi:10.3390/insects9010023)
Supplement: Supplementary file 1 [file insects-09-00023-s001.pdf]

insects

Article

A Point Mutation V419L in the Sodium Channel Gene from Natural Populations of *Aedes aegypti* Is Involved in Resistance to λ-Cyhalothrin in Colombia

Supplementary Materials:

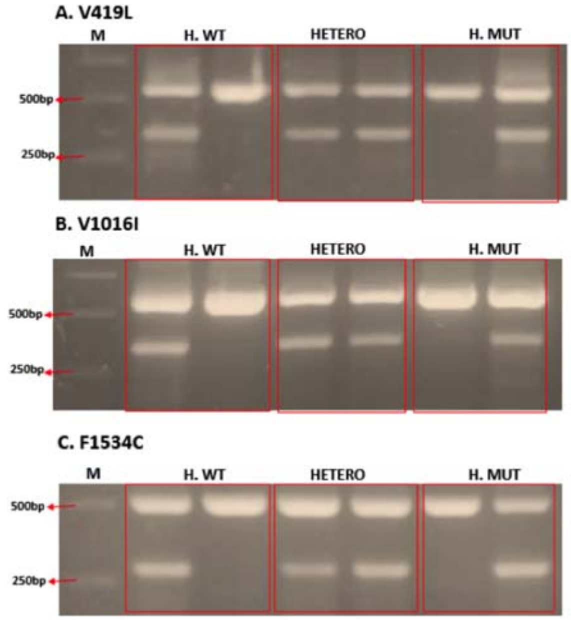

Figure S1. Allele specific PCR profile representative of point mutations obtained in *Ae. aegypti* mosquitoes from three Colombian cities. A. V419L. B. V1016I. C. F1534C. H.WT: Homozygote wild type, Hetero: heterozygote, H.MUT: homozygote mutated.

© 2018 by the authors. Submitted for possible open access publication under the terms and conditions of the Creative Commons Attribution (CC BY) license (http://creativecommons.org/licenses/by/4.0/).
